# IAA is more effective than EDTA in enhancing phytoremediation potential for cadmium and copper contaminated soils

**DOI:** 10.1186/s12870-024-05329-5

**Published:** 2024-08-29

**Authors:** Naila Shah, Muhammad Irshad, Waheed Murad, Muhammad Hamayun, Muhammad Qadir, Anwar Hussain, Hussan Ara Begum, Abdulwaahed Fahad Alrefaei, Mikhlid H. Almutairi, Ayaz Ahmad, Sajid Ali

**Affiliations:** 1 Department of Botany, Garden Campus, Abdul Wali Khan University Mardan, Mardan, Pakistan; 2 Department of Botany, Government Girls Degree College, Lundkhwar, Mardan Pakistan; 3 Department of Zoology, College of Science, King Saud University, Riyadh, Saudi Arabia; 4 Department of Biotechnology, Garden Campus, Abdul Wali Khan University Mardan, Mardan, Pakistan; 5 Department of Horticulture and Life Science, Yeungnam University, Gyeongsan, Republic of Korea

**Keywords:** Agricultural crops, Enhance phytoremediation, IAA, EDTA, Heavy metals, Polluted soil, Soil health

## Abstract

Enhanced phytoremediation offers a rapid and eco-friendly approach for cleaning agricultural soil contaminated with copper and cadmium which pose a direct threat to food scarcity and security. The current study aimed to compare the effectiveness of the two commonly used additives, IAA and EDTA, for the remediation of copper (Cu) and cadmium (Cd) contaminated soils using sunflower and maize. The plants were cultivated in pots under controlled conditions with four sets of treatments: control (0), Cu50/Cd50, Cu50/Cd50 + EDTA, and Cu50/Cd50 + IAA. The results showed that Cu50/Cd50 mg/kg drastically compromised the phytoremediation potential of both plants, as evident by reduced shoot and root length, and lower biomass. However, the augmentation of Cu50/Cd50 with EDTA or IAA improved the tested parameters. In sunflower, EDTA enhanced the accumulation of Cu and Cd by 58% and 21%, respectively, and improved plant biomass by 41%, compared to control treatment. However, IAA exhibited higher accumulation of Cu and Cd by 64% and 25%, respectively, and enhanced plant biomass by 43%. In case of maize, IAA was superior to EDTA which enhanced the accumulation of Cu and Cd by 87% and 32% respectively, and increased the plant biomass by 57%, compared to control treatment. Our findings demonstrate that foliar IAA is more effective than EDTA in enhancing the phytoremediation potential of sunflower and maize for Cu and Cd.

## Introduction

The modern world, due to its anthropogenic activities like urbanization and mechanistic agriculture leads to the accumulation of certain components in the soil like heavy metals (HMs) raises significant concerns [1, 2]. Among the heavy metals, cadmium (Cd) and copper (Cu) occupy important position. On one hand, the Cd serves no essential physiological or biochemical function in living organisms rather its elevated levels in soil can detrimentally affect plants, leading to symptoms such as chlorosis, inhibited growth, root tip browning, and ultimately, plant death [3]. On the other hand, copper (Cu) plays numerous vital roles in plants as aconstituent of various proteins and its involvement in ATP generation and CO_2_ assimilation during photosynthesis. However, excessive levels of Cu in ecosystems have arisen due to industrial and mining activities [4] that leads to physiological stress, impaired growth, and leaf chlorosis [5]. Owing to the high level of toxicity of these metals, it pose significant risks to human health when food with elevated level of these metals is consumed by them. Their continuous addition to the environment *via* industrial activities, agricultural practices, and improper waste management necessitates the development of efficient remediation techniques [3, 4]. In this connection, several remediation techniques are in place to remediate the soil and environment polluted with such heavy metals. However, most of these are based on chemical and physical treatment of the metals contaminated soils which are costly and less effective [6]. In contrast, phytoextraction as a green approach for removing soil toxins, has emerged to provide an eco-friendly, economic and sustainable solution to this environmental issue [7, 8]. Among the plants used for phytoremediation, plants with a short life cycle and faster growth rate effectively employed ton remove the potentially hazardous material such as heavy metals from polluted soils [9, 10]. These HMs are absorbed from the soil by roots and subsequently transmitted to shoots with differential selection that depend on the type of HM, plant species its growth stage and even the fertilizer used in the field [11].

Maize and sunflower are considered potentially effective to remediate HMs contaminate soils due to their high biomass production, fast growth rates, hyperaccumulator of HMs and tolerance to high concentrations of these HMs [8, 12–16]. Based on their potential to accumulate higher quantities of trace heavy metals, they can potentially be considered as hyperaccumulator especially for Cd, Cu, and Zn [17, 18]. Additionally, application of IAA and ethylenediaminetetraacetic acid (EDTA) can ameliorate the phytoremediation potential of these plants by improving the metal uptake and accumulation. EDTA reduces the pH of the soil by making complexes with HMs, improving bioavailability of HMs and empowering heavy metal stream from soil to roots and ultimately shoots [19, 20]. Likewise, the naturally occurring auxin (IAA) can protect plants from the detrimental effects exerted by heavy metals on their physiological and biochemical process [21–23]. Several other works have also depicted the role of EDTA and IAA for improving phytoextraction of different HMs by various plant species [14, 19]. However, none of these works has clearly outlined the comparative efficacy of EDTA and IAA as additives for improving the phytoremediation of HM-contaminated soils. This study is aimed to determine the differential efficacy of EDTA and IAA to extract Cu and Cd from artificially amended soils using maize and sunflower so that the treatment with better outcomes could be recommended for use in field.

## Materials and methods

### Experimental design and soil preparation

Healthy and viable sunflower (HYSUN-33) and maize (HYBRID P4040), seeds were obtained from the Agricultural Research Center (ARC) in Mardan, Pakistan. For surface sterilization, the seeds were soaked in 70% ethanol and then rinsed three times with distilled water. Filter paper assays were carried out to determine the effects of 50 mg/kg CdCl_2_ and CuCl_2_ each on seed germination following the methodology of CI Odoemena [24]. For the proficient growth of sunflower soil with a sandy loam texture was prepared by mixing sand (60%), silt (30%) and clay from the botanical garden (10%). The soil properties were checked and mentioned in Table 1.

**Table 1:** Experimental soil characteristics

Parameter	Value^a^	Units
Soil type	Sandy loam	
pH	7.74 ± 0.17^a^	
Relative humidity	50 to 60	%
Nitrogen	0.14 ± 0.003^a^	%
E/C	103 ± 0.023^a^	mili/S
Cadmium	1.27 ± 0.014^a^	mg/kg
Copper	12.2 ± 0.021^a^	mg/kg

The experiment was carried out in screen house with temperature range of 30–35 °C. Clay pots were filled with 5 kg of soil and were kept in the screen house. The screen house was rainproof, and the moisture level in the experimental soils was maintained at 60% of the WHC. The water holding capacity was measured by the weight difference of irrigated soil and oven dried soil. The pH of soil was measured by pH meter while electrical conductivity (EC) by EC meter. The experiment comprised selected metals, i.e., 50 mg/kg Cd and 50 mg/kg Cu, in combination. In this way, four broad sets of treatments were applied.

i) The positive control (plants without heavy metal stress).
ii) The negative control (plants treated only with cadmium or copper).
iii) Plants treated only with cadmium/copper and EDTA.
iv) Plants treated only with cadmium/copper and IAA.

**Fig. 1:**
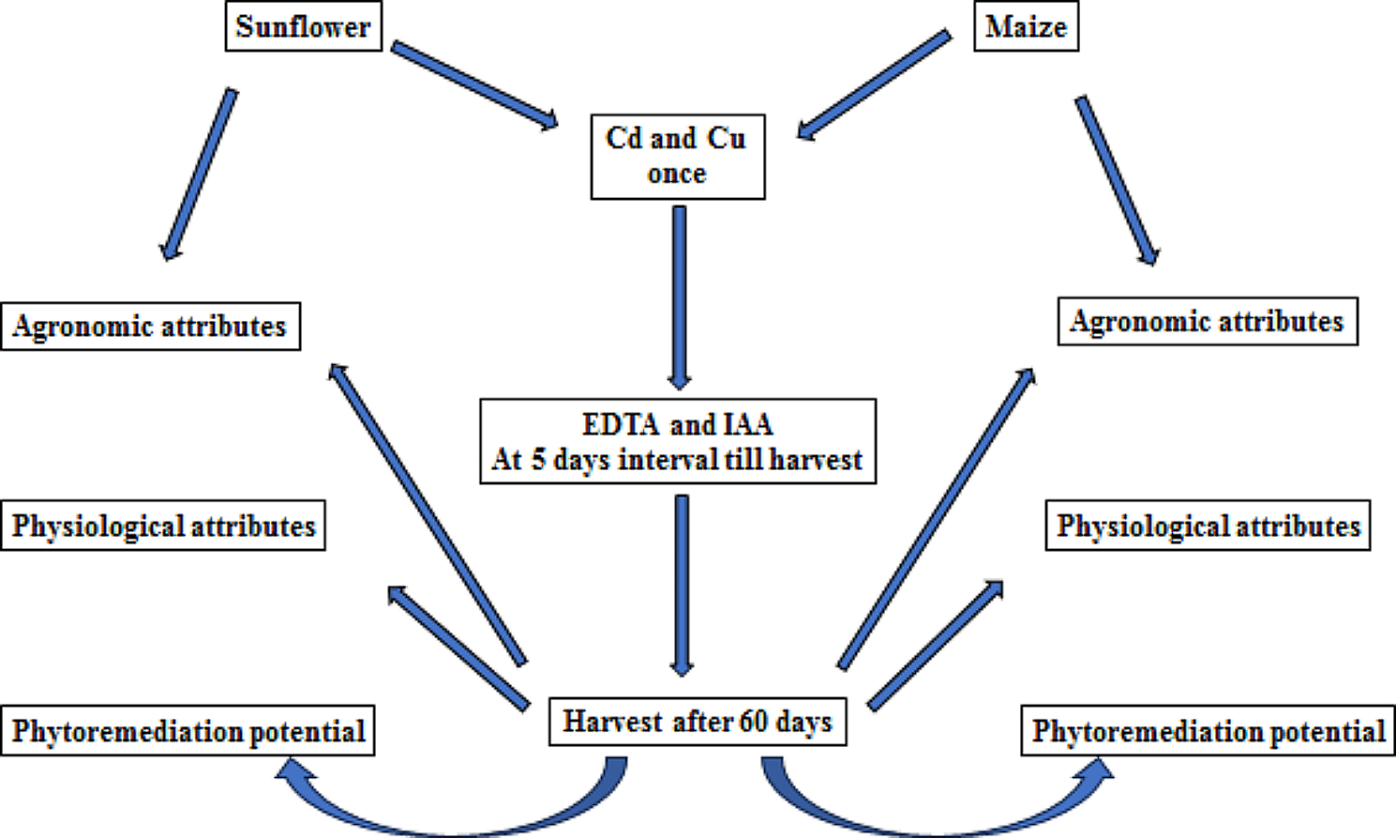
Schematic representation of the experimental setup

Each treatment comprised three replicates, with three plants in each. The selection of EDTA and IAA concentration was based on the available literature [11, 25, 26]. EDTA was added to the soil (5 mM), and IAA (100 mg/L) was sprayed foliarly starting from the 5th day of the metal and EDTA treatments and continued until the final harvest at an interval of 5 days. Pots were irrigated with tap water twice a day. After 60 days of growing the sunflower and maize plants under stressful conditions with proper care and observation, the plants were harvested and evaluated for the following parameters [14] as shown in Fig. 1.

### Morphological parameters

The agronomic features of sunflower and maize, including root and shoot length, were measured at each harvest. Plant height, fresh and dry mass of root and shoot was noted. In order to attain consistent weights of root and shoot tissues, they were kept for 48-hour duration and shade air dried before being subjected to an 80 °C oven drying process [26].

### Total chlorophyll contents

A standard protocol of DI Arnon [27] was followed to determine total chlorophyll contents in the leaves. Approximately, 0.5 gm fresh leaves of the host plant were homogenized in 80% acetone to extract the chlorophyll contents. The homogenate was further diluted by adding 7 mL of acetone (80%). The resultant solution was transferred to a separate sterile and clean vial and kept in a centrifuge machine for centrifugation at 5000–10,000 rpm for 5 min. Following that, the supernatant containing chlorophyll were separated in a clean separate vial, and repeated the same procedure until the pellet has no color. Using a spectrophotometer (Thermo, England), the optical density of the sample was determined at 645 nm and 663 nm versus 80% acetone (blank). The amounts of chlorophyll a and chlorophyll b determined using the formula below was added to determine the total chlorophyll content.

$$\text{C}\text{h}\text{l} \text{a}=\frac{ 12.3 \;O.D\left(663\right)-0.86 \;O.D\left(645\right)}{d \times 1000 \times w}\times V$$

$$\text{C}\text{h}\text{l} \text{b}=\frac{12.3 \;O.D\left(663\right)-0.86 \;O.D\left(645\right)}{d \times 1000 \times w}\times V$$

### Quantification of metals in plant biomass

Acid digestion procedures were used to assess the copper and cadmium contents of the plant biomass samples. For this purpose, acid digestion was performed on 0.5 g of oven-dried plant material using the ration of 4:1 of nitric acid (HNO_3_) and perchloric acid (HClO_4_) respectively [28]. The mixture was cooled to room temperature and then filtered through Whatman 42 filter paper. The final volume was adjusted to 25 mL by adding distilled water to the mixture. The same process was repeated for the control plants test, the solution was made using the same process as the sample solution, but no sample addition was employed. To evaluate the quantity of copper and cadmium in the samples, a flame atomic absorption spectroscope provided by Perkin Elmer (Analyst 700, built in the USA) was used. An acetylene/air flame was used to identify the required metal, and the manufacturer’s instructions were followed to during the measurements of the samples [29].

$$\begin{array}{l}{\rm{Remendiation}}\;{\rm{(\% )}}\\{\rm{ = }}\frac{{{\rm{Quantity}}\;{\rm{of}}\;{\rm{metal}}\;{\rm{accumulated}}\;{\rm{in}}\;{\rm{plants \times 100}}}}{{{\rm{Quantity}}\;{\rm{of}}\;{\rm{metal}}\;{\rm{applied}}\;{\rm{to}}\;{\rm{the}}\;{\rm{pots}}}}\end{array}$$

### Data analysis

The experiments were conducted six times under cadmium and copper treatment conditions, and SPSS Statistical Package version 21 was used to evaluate the results. The data from the experiments were analyzed using one-way ANOVA followed by Duncan Multiple Range Test (DMRT) for the calculation of significant difference at the level of *p* < 0.05 among the treatments (IBM, Armonk, NY, USA).

## Results

Phytoremediation effectiveness is primarily determined by two factors: the gathered biomass of hyper accumulator plants and the heavy metal concentrations in collected materials. As a result, in this work, the impacts of IAA and EDTA on the phytoremediation effectiveness of sunflower and maize are demonstrated in two ways: plant growth parameters and plant Cd and Cu absorption capacity.

### Effects of EDTA and IAA on the growth attributes of sunflower

**Fig. 2:**
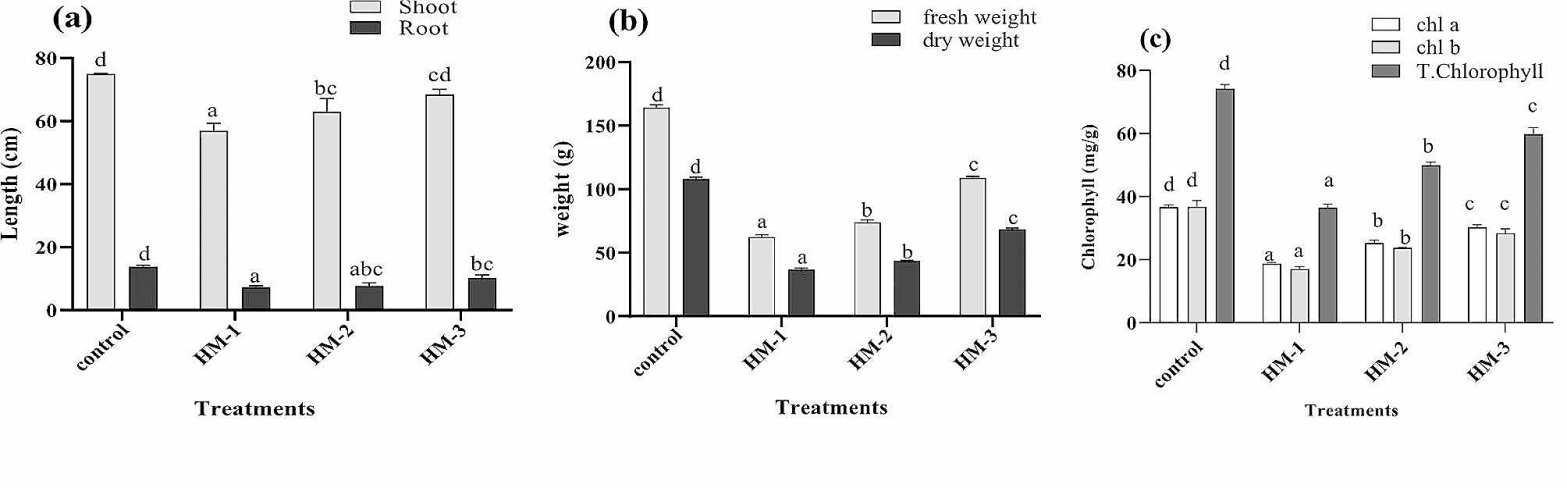
The effects of EDTA and IAA on the root/shoot length (**a**), fresh/dry weight (**b**) and total chlorophyll content (**c**) of sunflower plants exposed to the combined cadmium and copper stress. Bars represent the means of replicates with standard errors, and letters on the bars represent significance. (Control represents plants without any stress, HM-1 represents 50 mg/kg each of CdCl_2_ and CuCl_2_, HM-2 represents 50 mg/kg each of CdCl_2_ and CuCl_2_ with EDTA and HM-3 represents 50 mg/kg each of CdCl_2_ and CuCl_2_ with IAA

The effects of the concentrations of selected metal i.e., Cd and Cu as well as EDTA and IAA on the growth attributes of sunflower and maize in the present study are presented in (Fig. 2). The application of Cd and Cu in combination notably reduced the sunflower root shoot length by 47% and 24% respectively in comparison with those in the control (Fig. 2). However, the addition of EDTA with Cu and Cd improved sunflower root growth (8%) and shoot growth (2%). In addition, treatment with IAA, Cu and Cd showed positive effects on root growth, with an increase of 15%, and on shoot growth, with an increase of 21%, respectively.

The significant reductions in the fresh weight of sunflower plants in response to combined Cd and Cu stress were 51% and 65%, respectively. Sunflower plants treated with Cd, Cu and EDTA showed increase in fresh weight (28%) and dry weight (17%). Similarly, the Cd, Cu- and IAA treated sunflower plants showed a 25% increase in fresh weight and an 18% increase in dry weight (Fig. 2b). The concentrations of chlorophyll a and b and the total chlorophyll content decreased in response to combined Cd and Cu stress. However, the total chlorophyll content decreased by 49% with increasing concentrations of Cd and Cu, but the addition of EDTA resulted 14% increase in the total chlorophyll content. Moreover, chlorophyll degradation in the Cd and Cu treated sunflower plants significantly recovered (28%) after foliar application of IAA, which was also higher greater than the untreated control plants (Fig. 2c). Therefore, the application of EDTA and IAA play important roles in alleviating the negative effects of Cd and Cu stress on the growth of sunflower plants.

### Effects on the growth attributes of maize

**Fig. 3:**
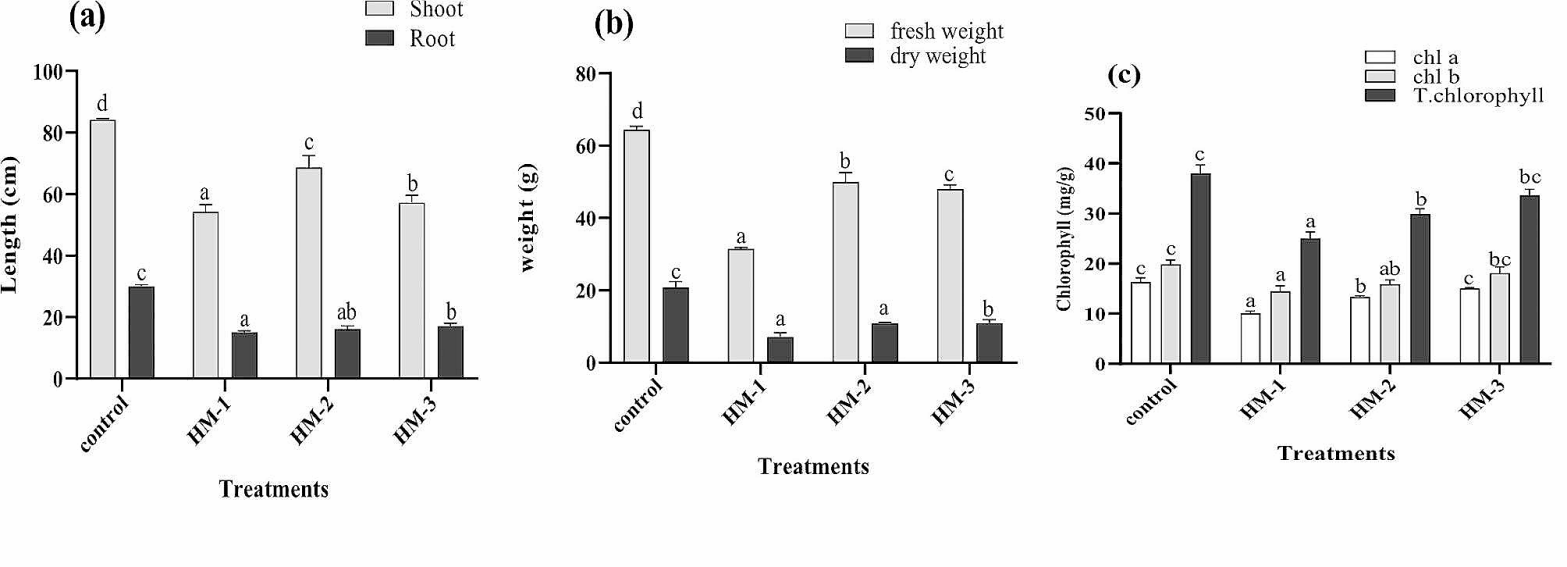
The effects of EDTA and IAA on the root/shoot length (**a**), fresh/dry weight (**b**), and total chlorophyll content (**c**) of maize plants exposed to the combined cadmium and copper stress

Maize plants were exposed to 50 mg/kg Cu or 50 mg/kg Cd. It reduced the plant height as well as the biomass of maize (Fig. 3a-b). In the presence of particular Cu and Cd metals in the soil, there was a 36% decrease in shoot growth compared to that of the control plants. A decrease in root length caused a decrease of 50%. The amendment of EDTA alone caused a change of 17% in the shoot and 3% in the root. During the application of IAA, little growth was recorded. A reduction in the fresh and dry weights of 61% and 65%, respectively, was recorded. Compared with those in the negative control treatment, the fresh and dry weights of maize plants in the presence of EDTA in the soil increased. However, significant increases of 28% and 29% were noted in response to the application of IAA. With the addition of a combination of the two metals, a decrease in the chlorophyll a, b, and total chlorophyll concentrations was observed. In maize, the overall chlorophyll concentration decreased by 30%. In comparison to those of the treated control, the total chlorophyll contents of the soil increased by 19% after the amendment of EDTA and by 25% after the solicitation of IAA by foliar spray (Fig. 3c).

### Bioaccumulation and biotranslocation of HM in sunflower

**Fig. 4:**
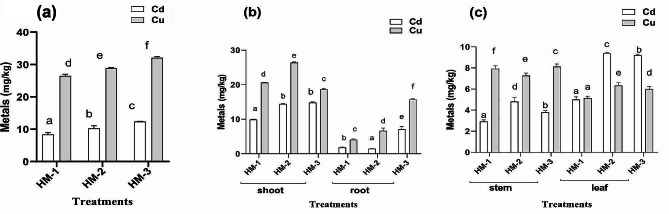
The effects of EDTA and IAA on metal accumulation in 60-day-old plants: (**a**) metal accumulation in roots and shoots (**b**) metal translocation in stems and leaves (**c**) in sunflower plants exposed to combined cadmium and copper stress

The use of a metal blend resulted in the bioaccumulation of copper and cadmium. As shown in Fig. 3a, the combined stress of both selected metals on sunflower was used as a negative control. Sunflower plants are hyperaccumulators of Cu and accumulate 53% of the total Cu and 16% of the total Cd from soil. In the sunflower plants, decreased cadmium accumulation was observed, although increased copper accumulation was noted. The use of EDTA in Cd- and Cu-treated plants resulted in a multifold increase in cadmium and copper accumulation, as Cu accumulation increased to 58%, and cadmium accumulation increased to 21%. Foliar IAA application combined with Cd and Cu increased the accumulation of Cu to 64% and cadmium to 25% (Fig. 4a). Similarly, the cadmium and copper ratios of the root-to-shoot ratio showed that the shoots of sunflower plants accumulated more cadmium and copper than did the roots. Metal accumulates in the roots and shoots when EDTA is applied to the soil and when IAA is applied foliage. Cd and Cu combined decreased Cd and increased Cu accumulation in the roots of sunflower plants. When the metals were mixed, a decrease in the amount of EDTA in the soil was documented. When the metal was supplemented as a mixture with the application of IAA as a foliar spray, Cd bioaccumulation decreased, but Cu bioaccumulation increased in the roots. Translocation of both metals was observed in the shoot (Fig. 4b). When the metal was added as a mixture, the opposite effect was observed, with stronger Cu biotranslocation and bioaccumulation and somewhat reduced Cd translocation and accumulation in the host plant shoots. When a combination of the chosen metals was added to the host plant soil, further gains were observed. For instance, the accumulation of cadmium and copper improved when the plants were treated with EDTA and foliar IAA. Contrary to expectations, in the case of leaves, the maximum amount of Cu accumulated compared to that of Cd, and the application of EDTA and IAA both increased the Cu concentration. In the case of stems, the maximum amount of Cu was present compared to that of cadmium. The application of EDTA increased cadmium concentrations in the stem while reducing copper. However, the application of IAA along with a blend of metals increased the amount of copper but decreased the amount of cadmium in the stems of sunflower plants (Fig. 4c).

### Bioaccumulation and biotranslocation of HM in maize

**Fig. 5:**
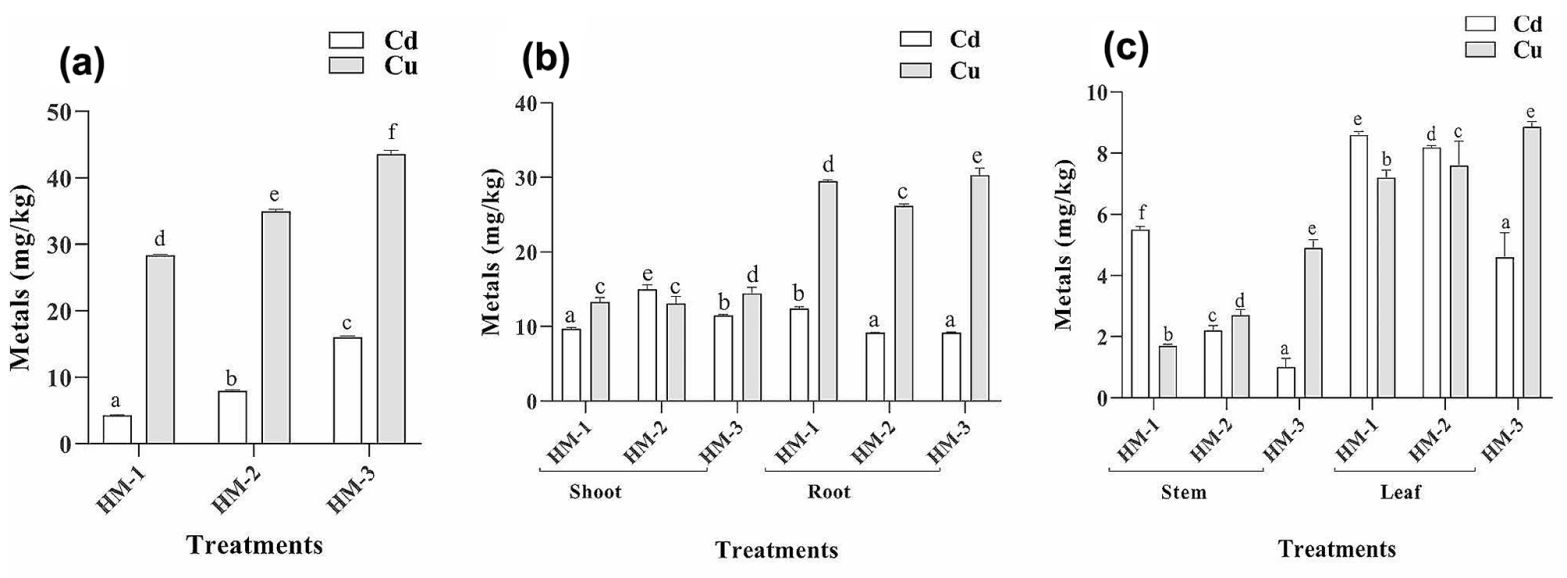
The effects of EDTA and IAA on metal accumulation in 60-day-old plants: (**a**) metal accumulation in roots and shoots, (**b**) metal translocation in stems and leaves, and (**c**) metal accumulation in maize exposed to combined cadmium and copper stress

The use of the specified metal combination causes a multiplication of cadmium and copper buildup. Maize plants have the ability to accumulate both metals to some extent. For instance, Cu accumulated at 56%, and cadmium accumulated at 9% in maize plants after 60 days of exposure to heavy metal stress. Compared to stress plants treated with both cadmium and copper, plants treated with both Cu and Cd and treated with EDTA exhibited increased absorption of both metals. The Cu accumulation increased to 70%, and the cadmium accumulation increased to 16%. In the same way, advantages were shown when foliar IAA and the contents of Cu and Cd in the bland were compared to those of plants subjected to stress control in which the mentioned metals were mixed. The application of IAA recovered copper accumulation by 87% and cadmium accumulation by 32% (Fig. 5a).

Similarly, when the amount of metals in the medium increased, so did the root-to-shoot cadmium ratio (Fig. 5b). The accumulation of Cd and Cu in maize shoots was nearly identical. In the case of roots, an intriguing outcome was reported. The roots had a greater concentration of copper than did the shoots of the maize plants. When Cu and Cd were supplemented as an amalgam with EDTA followed by foliar IAA, Cd accumulation decreased; however, Cu accumulation increased several-fold in the roots, with the greatest accumulation occurring in the roots. Compared to their respective control plants, plants treated with the metals in combination with EDTA and foliar IAA exhibited a further decrease in cadmium accumulation and a modest increase in copper accumulation (Fig. 5b). In the stems and leaves of maize plants, Cu and Cd are more abundant in the leaves than in the leaves. The exposure of the host to 50 mg/kg of Cu and Cd as a mixture resulted in the maximum accumulation of cadmium and lower bioaccumulation of copper in the leaves of the maize. The use of EDTA and IAA decreased Cd bioaccumulation in the stem and leaf but increased Cu accumulation in both parts of the maize plant (Fig. 5c).

### Effects EDTA and IAA on soil parameters

**Fig. 6:**
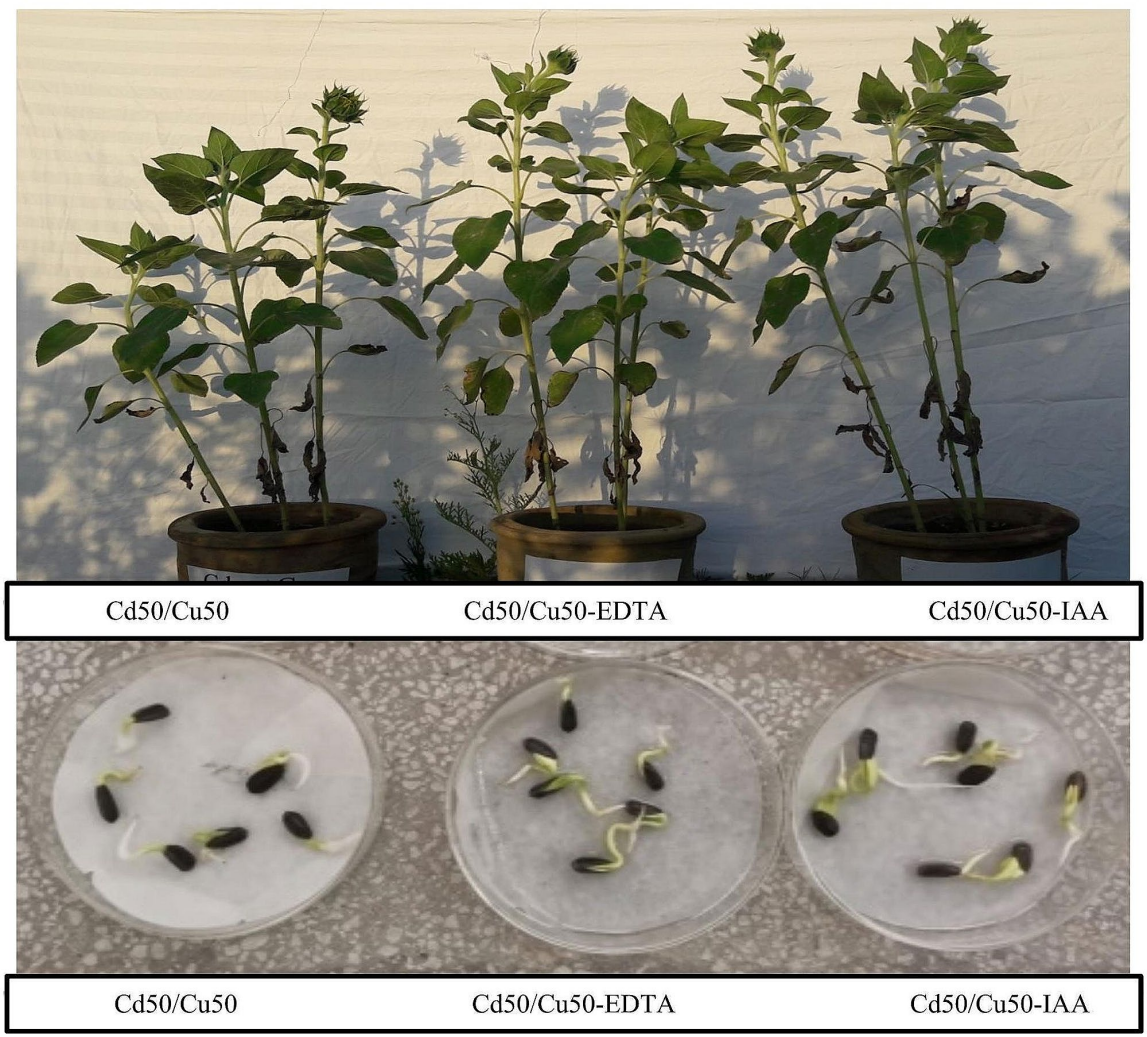
The effects of EDTA and IAA on germination and growth of sunflower in Cd/Cu stress

**Fig. 7:**
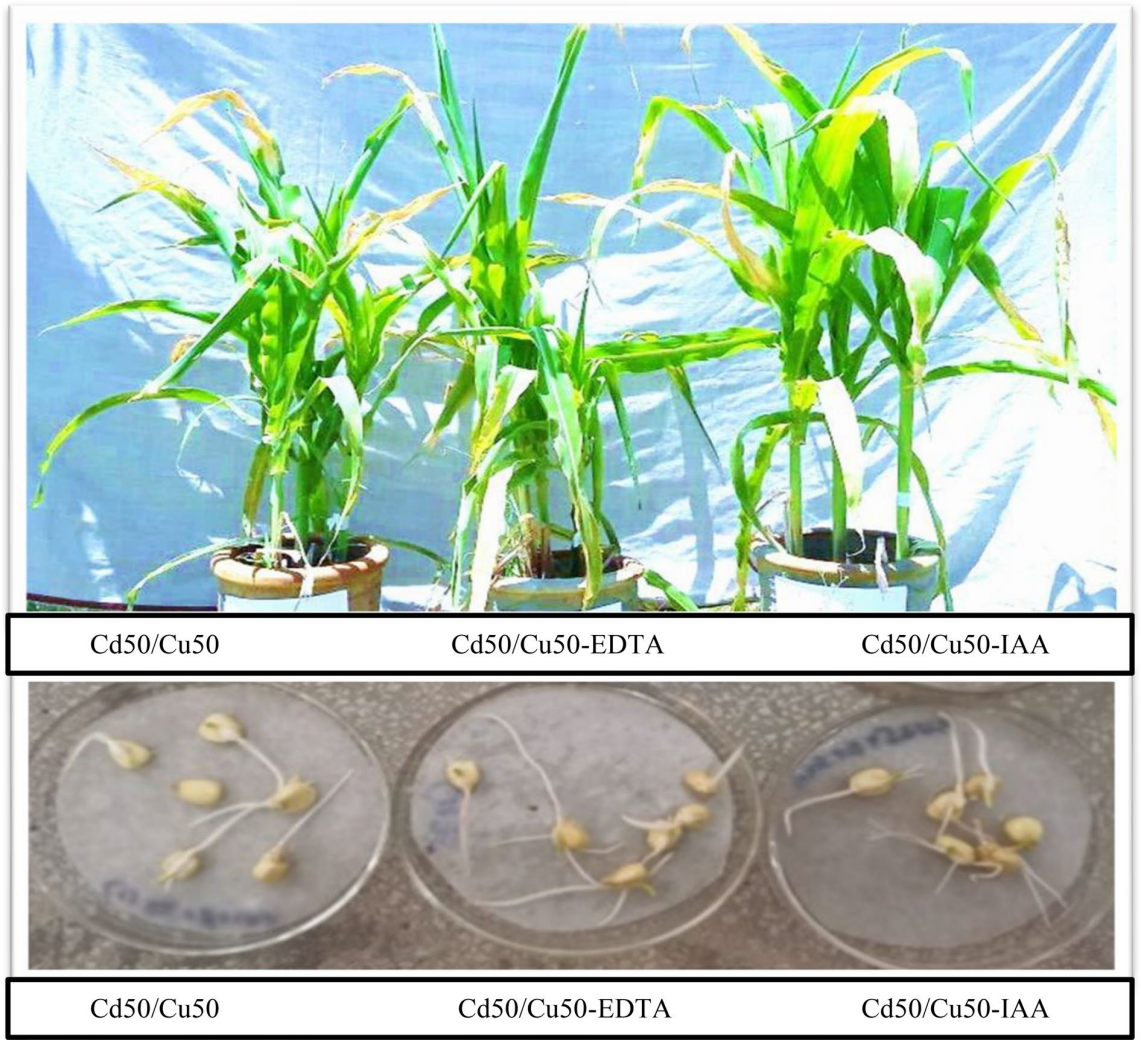
The effects of EDTA and IAA on germination and growth of sunflower in Cd/Cu stress

Soil parameters like pH and electrical conductivity are adversely affected by the combined stress of cadmium and copper-contaminated soil as compared to the positive control. However, the addition of EDTA to the soil improved the pH and electrical conductivity as compared to the negative control. The recovery of soil EC and pH is further strengthened by the foliar application of IAA (Table 2). Similarly, the remediation potential of sunflower and maize for Cd and Cu was determined separately. Sunflower has the potential to remediate cadmium to 17% and copper to 53%, respectively, without EDTA or IAA. The remediation percentage was enhanced to 21% for cadmium and 58% for copper. With the application of foliar spray, the percentage of remediation was marked at 25% for Cd and 64% for Cu. Maize has the potential to remediate cadmium to 9% and copper to 5%, respectively, without any additive. The remediation percentage was enhanced to 16% for cadmium and 70% for copper. With the application of foliar spray, the percentage remediation was marked at 32% for Cd and 87% for Cu (Table 3). Moreover, EDTA and IAA were applied to sunflower and maize plants grown under Cd and Cu stress. As a result, no significant effect on the germination of either sunflower or maize was detected (Figs. 6 and 7). The other parameters were variably affected by the selected metals, EDTA and IAA, to different extents.

**Table 2:** Effects of EDTA and IAA on electrical conductivity and pH of soil. Where (±) represent standard error and different letters for standard deviation

Treatments	E/C (miliS/cm)		pH	
	Sunflower	Maize	Sunflower	Maize
Control	88 ± 0.43^a^	97 ± 2.23^a^	7.7 ± 1.43^a^	7.8 ± 0.12^a^
Cd/Cu	100 ± 1.40^b^	137 ± 2.02^c^	8.1 ± 0.09^b^	8.1 ± 1.02^b^
Cd/Cu + EDTA	109 ± 1.35^c1^	98 ± 1.35^b^	8.0 ± 0.80^b^	8.0 ± 0.75^b^
Cd/Cu + IAA	101 ± 1.22^b^	92 ± 1.27^a^	7.8 ± 0.36^a^	8.0 ± 0.60^b^

**Table 3:** Effect of EDTA and IAA on the Phytoremediation (%) potential of sunflower and maize

Treatments	Phytoremediation (%)			
	Sunflower		Maize	
	**Cd**	**Cu**	**Cd**	**Cu**
Cd/Cu	17 ± 0.12^a^	53 ± 2.02^a^	9 ± 1.34^a^	57 ± 1.03^a^
Cd/Cu + EDTA	21 ± 1.34^b^	58 ± 2.01^b^	16 ± 0.07^b^	70 ± 2.04^b^
Cd/Cu + IAA	25 ± 2.45^c^	64 ± 1.02^c^	32 ± 0.9^c^	87 ± 1.25^c^

## Discussion

Soil is an important abiotic component of ecosystem that supports life on earth and regulates nutrient cycling. However, the mechanistic agriculture and industrialization cause soil pollution. The deleterious effects of high concentrations of copper and cadmium on the growth and biomass of maize and sunflower plants have been studied, as reported previously [6, 8, 14, 30, 31]. A high Cu content in soil can induce nutritional imbalances, reducing plant productivity and agricultural output [32]. The plant growth and biomass reduction of sunflower and maize at high concentrations of Cu in the soil might be attributed to high Cu deposition in various parts of plants, resulting in phytotoxic effects [33–36]. Due to the potential formation of reactive oxygen species in metal-exposed maize, a greater Cd concentration in the soil may have an adverse effect on photosynthetic pigments, plant development, and vintage. Higher levels of cadmium in maize, which reduces chlorophyll contents in leaves, may be responsible for the lower biomass [14, 37].

Cadmium and copper assimilation and biotranslocation to various plants parts growing in HM-polluted conditions metal availability and host growth attributes [11, 21, 33]. The information on the take-up of copper and cadmium by maize and sunflower varied. Being an essential micronutrient, plant have special channels for the absorption of Cu, and easily be absorbed by the host plant immediately, while cadmium take-up is relatively slower as cadmium is non-essential heavy metal and plant have no specific carriers and channels for their absorption. Sunflower plants are hyperaccumulators of both cadmium and Cu, while maize plants buildup copper levels the most copper in their below ground parts, and generally trace amount of copper is moved to above ground parts of the host plants as per their requirement of the metal [7, 38]. Similar results were also recorded in case of chromium and arsenic in which higher IAA producing rhizobacteria triggered the sunflower to accumulate chromium and arsenic in their underground parts [39, 40]. It is thought that the limited ability of plants to ingest various metals in the aforementioned region may be due to their tolerance to metal stress. Cu is substantially concentrated in the root, but only a small quantity is transported to the shoot [38]. However, in the case of sunflower, the results are not the same. Sunflower strongly hyperaccumulates Cu in shoots rather than in roots [30]. The maximum amount of cadmium is present in the shoots of maize, while little cadmium is present in the roots [41]. The reason behind the distribution of the Cu was to compartmentalize the metal and lower their potential toxicity [42].

According to different studies, both EDTA and IAA are used to increase the Cu and Cd phytoremediation potential of sunflower and maize [8, 14, 19, 26, 43]. EDTA significantly improved plant biomass and growth in *Dianthus chinensis, Zea mays, Chlorophytum comosum and Helianthus annuus* [8, 44, 45]. The increase in biomass and growth may be due to improved gaseous exchange [11]. Another reason may be that EDTA chelates metals, reduces toxicity and promotes growth [46, 47]. As proposed by Kanwal [48], EDTA induces the chelation of metal ions and reduces free metal ions in plants. EDTA amendment to the soil further increased the concentrations of Cu and Cd in the shoots and roots of sunflower and maize plants. The increased amount of both cadmium and copper may be due to chelation of metal with EDTA. The increase of metal accumulation with EDTA and IAA blend was probably due to their action is in two folds i.e., IAA increase the surface are of the root providing more access to the nutrient in the soil while EDTA making chelate to prevent leaching of these metal and hence can be acquired by the host plant from the soil. Our results are comparable to those of Jan et al. [49] who reported that the introduction of EDTA, IAA and GA helps *Dysphania ambrosioides (L.) Mosyakin & Clemants* to accumulate higher quantities of Cd while growing normally. The addition of EDTA to soil enhanced the accumulation of Pb in the roots of *Vetiveria zizanioides* [50], *Sedum* [51], *and Vicia faba* seedlings [19] and in the shoots of *Canavalia ensiformis* L. [52]. When lead and EDTA were applied to a hydroponically grown sunflower, the amount of lead accumulation increased, as in the case of Cd accumulation in the present study [53]. Following EDTA treatment, Pb, Cd, and Zn accumulation increases in a range of plant species from several families, most notably *Cannabis sativa*, *Zea mays*, *Sorghum vulgare*, and *Medicago sativa*. It has been reported that EDTA has the potential to enhance the hyperaccumulation of HMs, particularly in nonhyperaccumulator families [54].

It has been widely documented that IAA can boost plant root and shoot growth under salt and heavy metal stress [26, 55–57]. Moreover, different molecular examinations have shown that auxin synthesis and signaling are vigorously dependent on a few genes related with auxin synthesis that are stimulated because of HM toxic effects [58]. For example, in comparison with control plants, maize exposed to Cd polluted soils within the presence of IAA showed higher root development and extension [59]. Essentially, foliar auxin (IAA) administration enhanced the total plant biomass of sunflower plants filling in Cd/Cu-polluted soil [60]. To neutralize the unfavorable effects of HM, it has been uncovered that auxin formation, i.e., IAA-Asp, changes CAT and POD action, advances carboxylation of protein, and diminishes H_2_O_2_ and MDA contents [61, 62].

## Conclusion

Previous studies revealed that cadmium and copper, when present at the level of 50 mg/kg negatively affect the development, biomass production, and total chlorophyll content of sunflower and maize plants. EDTA supplementation of Cu and Cd-contaminated soils and the foliar application of IAA significantly enhanced the phytoremediation potential of sunflower and maize which exhibited improved morphological parameters in both plants. However, IAA was superior to EDTA in terms of heavy metals accumulation and enhancing biomass and plant length, especially in sunflower plants. Our results demonstrate optimizing concentrations IAA as effective additive for enhanced phytoremediation potential of plants, without compromising their normal physiological and morphological features.

## Data Availability

Data is provided within the manuscript or supplementary information files.

## References

[1] Amouei A, Cherati A, Naghipour D. Heavy metals contamination and risk assessment of surface soils of Babol in northern Iran. Health Scope 2018, 7(1).

[2] Khan A, Khan MS, Egozcue JJ, Shafique MA, Nadeem S, Saddiq G. Irrigation suitability, health risk assessment and source apportionment of heavy metals in surface water used for irrigation near marble industry in Malakand, Pakistan. PLoS ONE. 2022;17(12):e0279083. 36542623 10.1371/journal.pone.0279083 PMC9770375

[3] Asati A, Pichhode M, Nikhil K. Effect of heavy metals on plants: an overview. Int J Application or Innov Eng Manage. 2016;5(3):56–66.

[4] Roy RN, Finck A, Blair G, Tandon H. Plant nutrition for food security. Guide Integr Nutrient Manage FAO Fertilizer Plant Nutr Bull. 2006;16(368):201–14.

[5] Khator K, Shekhawat G. Cd-and Cu-induced phytotoxicity on 2–3 leaf stage of Cyamopsis tetragonoloba and its regulation by nitrate reductase and ROS quenching enzyme. Acta Physiol Plant. 2020;42(7):120. 10.1007/s11738-020-03105-0

[6] Liu Y-M, Liu D-Y, Zhang W, Chen X-X, Zhao Q-Y, Chen X-P, Zou C-Q. Health risk assessment of heavy metals (Zn, Cu, Cd, Pb, as and cr) in wheat grain receiving repeated zn fertilizers. Environ Pollut. 2020;257:113581. 31753641 10.1016/j.envpol.2019.113581

[7] Bhat SA, Bashir O, Haq SAU, Amin T, Rafiq A, Ali M, Américo-Pinheiro JHP, Sher F. Phytoremediation of heavy metals in soil and water: an eco-friendly, sustainable and multidisciplinary approach. Chemosphere. 2022;303:134788. 35504464 10.1016/j.chemosphere.2022.134788

[8] Shah N, Qadir M, Irshad M, Hussain A, Hamayun M, Murad W, Khan A, Al-Harrasi A. Enhancement of Cadmium Phytoremediation potential of Helianthus annuus L. with application of EDTA and IAA. Metabolites. 2022;12(11):1049. 36355131 10.3390/metabo12111049 PMC9692325

[9] Elekes CC. Eco-technological solutions for the remediation of polluted soil and heavy metal recovery. Environ Risk Assess Soil Contam InTech Rijeka 2014:309–35.

[10] Khan IU, Qi S-S, Gul F, Manan S, Rono JK, Naz M, Shi X-N, Zhang H, Dai Z-C, Du D-L. A green approach used for heavy metals ‘phytoremediation’via invasive plant species to mitigate environmental pollution: a review. Plants. 2023;12(4):725. 36840073 10.3390/plants12040725 PMC9964337

[11] Saleem MH, Ali S, Kamran M, Iqbal N, Azeem M, Tariq Javed M, Ali Q, Zulqurnain Haider M, Irshad S, Rizwan M. Ethylenediaminetetraacetic acid (EDTA) mitigates the toxic effect of excessive copper concentrations on growth, gaseous exchange and chloroplast ultrastructure of Corchorus capsularis L. and improves copper accumulation capabilities. Plants. 2020;9(6):756. 32560128 10.3390/plants9060756 PMC7356174

[12] Jan FA, Saleem S, Faisal S, Hussain I, Rauf A, Ullah N. Road dust as a useful tool for the assessment of pollution characteristics and health risks due to heavy metals: a case study from District Charsadda, Pakistan. Arab J Geosci. 2021;14:1–13. 10.1007/s12517-021-08342-2

[13] Kimenyu P, Oyaro N, Chacha J, Tsanuo M. The potential of Commelina bengalensis, Amaranthus hybridus, Zea mays for phytoremediation of heavy metals from contaminated soils. 2009.

[14] Shah N, Irshad M, Hussain A, Mehmood A, Murad W, Qadir M, Awais M, Shah M, Khan N. The deteriorating effects of cadmium accumulation on the yield and quality of maize crop. South Afr J Bot. 2023;160:732–8. 10.1016/j.sajb.2023.07.050

[15] Chirakkara RA, Cameselle C, Reddy KR. Assessing the applicability of phytoremediation of soils with mixed organic and heavy metal contaminants. Reviews Environ Sci Bio/Technology. 2016;15:299–326. 10.1007/s11157-016-9391-0

[16] Zalewska M, Nogalska A. Phytoextraction potential of sunflower and white mustard plants in zinc-contaminated soil. Chil J Agricultural Res. 2014;74(4):485–9. 10.4067/S0718-58392014000400016

[17] Wuana R, Okieimen F, Imborvungu J. Removal of heavy metals from a contaminated soil using organic chelating acids. Int J Environ Sci Technol. 2010;7:485–96. 10.1007/BF03326158

[18] Chigbo C, Batty L. Chelate-assisted phytoremediation of Cu-pyrene-contaminated soil using Z. mays. Water Air Soil Pollut. 2015;226:1–10. 10.1007/s11270-014-2277-2

[19] Shahid M, Pourrut B, Dumat C, Nadeem M, Aslam M, Pinelli E. Heavy-metal-induced reactive oxygen species: phytotoxicity and physicochemical changes in plants. Reviews Environ Contam Toxicol. 2014;232:1–44. 10.1007/978-3-319-06746-9_1 24984833

[20] Awokunmi E. The potential of Abelmoschus esculentus in EDTA-assisted phytoextraction of heavy metals from soil of bashiri dumpsite, Ado Ekiti, Nigeria. Int J Environ Prot. 2016;6(1):72–7.

[21] Rahman SU, Han J-C, Zhou Y, Ahmad M, Li B, Wang Y, Huang Y, Yasin G, Ansari MJ, Saeed M. Adaptation and remediation strategies of mangroves against heavy metal contamination in global coastal ecosystems: a review. J Clean Prod 2024:140868.

[22] Dutta N, Dutta S, Bhupenchandra I, Karmakar R, Das K, Singh LK, Bordoloi A, Sarmah T. Assessment of heavy metal status and identification of source in soils under intensive vegetable growing areas of Brahmaputra valley, North East India. Environ Monit Assess. 2021;193:1–18. 10.1007/s10661-021-09168-x 34075481

[23] Hemantaranjan A, Katiyar D, Vyas J, Bhanu AN. Alleviating effect of foliar sprayed paclobutrazol and salicylic acid on biochemical changes in mungbean under cadmium stress. J Plant Sci Res. 2018;34(1):37–44. 10.32381/JPSR.2018.34.01.5

[24] Odoemena CI. Influence of poultry manure amendment on bioremedial activity of petroleum oil polluted soil and early cowpea growth. Global J Agricultural Sci. 2002;1(1):41–7.

[25] Jin H, Zhihong P, Jiaqing Z, Chuxuan L, Lu T, Jun J, Xinghua L, Wenyan G, Junkang G, Binbin S. Source apportionment and quantitative risk assessment of heavy metals at an abandoned zinc smelting site based on GIS and PMF models. J Environ Manage. 2023;336:117565. 36868153 10.1016/j.jenvman.2023.117565

[26] Ji W, Yang Z, Yu T, Yang Q, Wen Y, Wu TJBEC. Toxicology: potential ecological risk assessment of heavy metals in the Fe–Mn nodules in the karst area of Guangxi, Southwest China. 2021, 106:51–6. 10.1007/s00128-020-02837-6 32239254

[27] Arnon DI. Copper enzymes in isolated chloroplasts. Polyphenoloxidase in Beta vulgaris. Plant Physiol. 1949;24(1):1. 16654194 10.1104/pp.24.1.1 PMC437905

[28] Allen SE, Grimshaw HM, Parkinson JA, Quarmby C. Chemical analysis of ecological materials; 1974.

[29] Zahoor M, Irshad M, Rahman H, Qasim M, Afridi SG, Qadir M, Hussain A. Alleviation of heavy metal toxicity and phytostimulation of Brassica campestris L. by endophytic Mucor sp. MHR-7. Ecotoxicol Environ Saf. 2017;142:139–49. 28407499 10.1016/j.ecoenv.2017.04.005

[30] Jadia CD, Fulekar MH. Phytoremediation: the application of vermicompost to remove zinc, cadmium, copper, nickel and lead by sunflower plant. Environ Eng Manag J 2008, 7(5).

[31] Rizwan M, Ali S, Ali B, Adrees M, Arshad M, Hussain A, ur Rehman MZ, Waris AA. Zinc and iron oxide nanoparticles improved the plant growth and reduced the oxidative stress and cadmium concentration in wheat. Chemosphere. 2019;214:269–77. 30265934 10.1016/j.chemosphere.2018.09.120

[32] Rashid A, Ryan J. Micronutrient constraints to crop production in soils with Mediterranean-type characteristics: a review. J Plant Nutr. 2004;27(6):959–75. 10.1081/PLN-120037530

[33] Nagajyoti PC, Lee KD, Sreekanth TJE. Heavy metals, occurrence and toxicity for plants: a review. 2010, 8:199–216.

[34] Sharma RK, Agrawal MJJB. Biological effects of heavy metals: an overview. 2005, 26(2):301–13. 16334259

[35] Thounaojam TC, Panda P, Mazumdar P, Kumar D, Sharma GD, Sahoo L. Sanjib PJPp, biochemistry: excess copper induced oxidative stress and response of antioxidants in rice. 2012, 53:33–9. 10.1016/j.plaphy.2012.01.006 22306354

[36] Zvezdanović J, Cvetić T, Veljović-Jovanović S, Marković DJRP, Chemistry. Chlorophyll bleaching by UV-irradiation in vitro and in situ: absorption and fluorescence studies. 2009, 78(1):25–32.

[37] Baryla A, Carrier P, Franck F, Coulomb C, Sahut C, Havaux MJP. Leaf chlorosis in oilseed rape plants (Brassica napus) grown on cadmium-polluted soil: causes and consequences for photosynthesis and growth. 2001, 212:696–709. 10.1007/s004250000439 11346943

[38] Niazy MM, Wahdan MJZJoAR, ENHANCING PHYTOREMEDIATION OF Pb BY TREATING SOIL WITH CITRIC ACID AND GROWING WHITE JUTE. (Corchorus capsularis, L.), AND RIVER RED GUM (Eucalyptus camaldulensis). 2017, 44(4):1359–67.

[39] Qadir M, Hussain A, Hamayun M, Shah M, Iqbal A, Husna, Murad W. Phytohormones producing rhizobacterium alleviates chromium toxicity in *Helianthus annuus* L. by reducing chromate uptake and strengthening antioxidant system. Chemosphere. 2020;258:127386. 32559495 10.1016/j.chemosphere.2020.127386

[40] Qadir M, Hussain A, Shah M, Hamayun M, Iqbal A, Irshad M, Khan ZH, Islam B, Elansary HO, Mahmoud EA. *Pantoea conspicua* promoted sunflower growth and engulfed rhizospheric arsenate by secreting exopolysaccharide. Plant Physiol Biochem 2023:107826. 10.1016/j.plaphy.2023.107826 37331076

[41] Lukačová Kuliková Z. Lux AJBoEC, Toxicology: Silicon influence on maize, Zea mays L., hybrids exposed to cadmium treatment. 2010, 85:243–50. 10.1007/s00128-010-0046-5 20563865

[42] Roméo M, Gnassia-Barelli M. Metal distribution in different tissues and in subcellular fractions of the Mediterranean clam ruditapes decussatus treated with cadmium, copper, or zinc. Comp Biochem Physiol Part C: Pharmacol Toxicol Endocrinol. 1995;111(3):457–63.

[43] Chen L, Zhang X, Zhang M, Zhu Y, Zhuo RJJ. Removal of heavy-metal pollutants by white rot fungi: mechanisms, achievements, and perspectives. 2022, 354:131681.

[44] Wang S-L, Xu X-R, Sun Y-X, Liu J-L, Li H-BJM. Heavy metal pollution in coastal areas of South China: a review. 2013, 76(1–2):7–15. 10.1016/j.marpolbul.2013.08.025 24084375

[45] Suthar V, Memon KS. Mahmood-Ul-Hassan MJEm, assessment: EDTA-enhanced phytoremediation of contaminated calcareous soils: heavy metal bioavailability, extractability, and uptake by maize and sesbania. 2014, 186:3957–68. 10.1007/s10661-014-3671-3 24515546

[46] Habiba U, Ali S, Farid M, Shakoor MB, Rizwan M, Ibrahim M, Abbasi GH, Hayat T, Ali BJES, Research P. EDTA enhanced plant growth, antioxidant defense system, and phytoextraction of copper by Brassica napus L. 2015, 22:1534–44. 10.1007/s11356-014-3431-5 25163559

[47] Lambrechts L, Paaijmans KP, Fansiri T, Carrington LB, Kramer LD, Thomas MB. Scott TWJPotNAoS: impact of daily temperature fluctuations on dengue virus transmission by Aedes aegypti. 2011, 108(18):7460–5. 10.1073/pnas.1101377108 PMC3088608 21502510

[48] Kanwal A, Ali S, Farhan MJI. Heavy metal phytoextraction potential of indigenous tree species of the family fabaceae. 2019, 21(3):251–8. 10.1080/15226514.2018.1524828 30656955

[49] Jan, et al. Plant growth regulators and EDTA improve phytoremediation potential and antioxidant response of Dysphania ambrosioides (L.) Mosyakin & Clemants in a Cd-spiked soil. Environ Sci Pollut Res. 2021;28:43417–30. 10.1007/s11356-021-13772-2 33830421

[50] Andra SS, Datta R, Sarkar D, Saminathan SK, Mullens CP, Bach SBJEP. Analysis of phytochelatin complexes in the lead tolerant vetiver grass [Vetiveria zizanioides (L.)] using liquid chromatography and mass spectrometry. 2009, 157(7):2173–83. 10.1016/j.envpol.2009.02.014 19282075

[51] Sun Y-b, Zhou Q-x, An J, Liu W-t, Liu RJG. Chelator-enhanced phytoextraction of heavy metals from contaminated soil irrigated by industrial wastewater with the hyperaccumulator plant (Sedum Alfredii Hance). 2009, 150(1–2):106–12.

[52] Gabos MB, Abreu CAd, Coscione ARJSA. EDTA assisted phytorremediation of a pb contamined soil: metal leaching and uptake by Jack beans. 2009, 66:506–14.

[53] Seth CS, Misra V, Chauhan LJI. Accumulation, detoxification, and genotoxicity of heavy metals in Indian mustard (Brassica juncea L.). 2012, 14(1):1–13. 10.1080/15226514.2011.555799 22567690

[54] Souza LA, Piotto FA, Nogueirol RC, Azevedo RA. Use of non-hyperaccumulator plant species for the phytoextraction of heavy metals using chelating agents. Scientia Agricola. 2013;70:290–5. 10.1590/S0103-90162013000400010

[55] Agami RA, Mohamed GFJE. Safety e: exogenous treatment with indole-3-acetic acid and salicylic acid alleviates cadmium toxicity in wheat seedlings. 2013, 94:164–71. 10.1016/j.ecoenv.2013.04.013 23684274

[56] Hadi F, Bano A, Fuller MPJC. The improved phytoextraction of lead (pb) and the growth of maize (Zea mays L.): the role of plant growth regulators (GA3 and IAA) and EDTA alone and in combinations. 2010, 80(4):457–62. 10.1016/j.chemosphere.2010.04.020 20435330

[57] Qadir M, Hussain A, Shah M, Lee IJ, Iqbal A, Irshad M, Ismail, Sayyed A, Husna, Ahmad A, et al. Comparative assessment of chromate bioremediation potential of *Pantoea conspicua* and *Aspergillus Niger*. J Hazard Mater. 2022;424:127314. 34600376 10.1016/j.jhazmat.2021.127314

[58] Fattorini L, Ronzan M, Piacentini D, Della Rovere F, De Virgilio C, Sofo A, Altamura M, Falasca GJE. Botany e: cadmium and arsenic affect quiescent centre formation and maintenance in Arabidopsis thaliana post-embryonic roots disrupting auxin biosynthesis and transport. 2017, 144:37–48.

[59] Tahir MB, Kiran H, Iqbal TJES, Research P. The detoxification of heavy metals from aqueous environment using nano-photocatalysis approach: a review. 2019, 26(11):10515–28. 10.1007/s11356-019-04547-x 30835072

[60] Liphadzi M, Kirkham MJSAJB. Availability and plant uptake of heavy metals in EDTA-assisted phytoremediation of soil and composted biosolids. 2006, 72(3):391–7.

[61] Srivastava V, Sarkar A, Singh S, Singh P, De Araujo AS. Singh RPJFiES: agroecological responses of heavy metal pollution with special emphasis on soil health and plant performances. 2017, 5:64.

[62] Jaouani K, Karmous I, Ostrowski M, El Ferjani E, Jakubowska A, Chaoui, AJJopp. Cadmium effects on embryo growth of pea seeds during germination: investigation of the mechanisms of interference of the heavy metal with protein mobilization-related factors. 2018, 226:64–76. 10.1016/j.jplph.2018.02.009 29704645

